# Caregiver satisfaction with early integrated palliative care in oncology: secondary outcomes from the PALLiON cluster-RCT

**DOI:** 10.3389/fonc.2026.1787814

**Published:** 2026-06-18

**Authors:** Kristin Vassbotn Guldhav, Andrew Athan McAleavey, Nina Aass, Sigve Andersen, Bente Mirjam Christensen, Olav Dajani, Kari Eldal, Nienke Aafke de Glas, Herish Garresori, Hanne Hamre, Ellinor Christin Haukland, Pål Andre Hegland, Tonje Lundeby, Erik Torbjørn Løhre, Ørnulf Paulsen, Torunn Wester, Stein Kaasa, Marianne Jensen Hjermstad, John Roger Andersen

**Affiliations:** 1 Department of Oncology, Førde Central Hospital, Førde, Norway; 2 Faculty of Health and Social Sciences, Department of Health and Function, Western Norway University of Applied Sciences, Bergen, Norway; 3 Faculty of Health and Social Sciences, Department of Health and Caring Sciences, Western Norway University of Applied Sciences, Førde, Norway; 4 Center for Health Research, Førde Hospital Trust, Førde, Norway; 5 European Palliative Care Research Centre (PRC), Department of Oncology, Oslo University Hospital, and Institute of Clinical Medicine, University of Oslo, Oslo, Norway; 6 Regional Advisory Unit in Palliative Care, Department of Oncology, Oslo University Hospital, Oslo, Norway; 7 Regional Advisory Unit in Palliative Care, Department of Oncology, University Hospital of North Norway, Tromsø, Norway; 8 Institute of Clinical Medicine, UiT, The Arctic University of Norway, Tromsø, Norway; 9 Center for Cancer Treatment, Sorlandet Hospital, Kristiansand, Norway; 10 Department of Hematology and Oncology, Stavanger University Hospital, Stavanger, Norway; 11 Department of Oncology, Akershus University Hospital, Lørenskog, Norway; 12 Department of Oncology and Palliative Care, Nordland Hospital Trust, Bodø, Norway; 13 Cancer Clinic, St. Olavs Hospital, Trondheim University Hospital, Trondheim, Norway; 14 Department of Clinical and Molecular Medicine, Faculty of Medicine and Health Sciences, NTNU, Norwegian University of Science and Technology, Trondheim, Norway; 15 Palliative Care Unit, Telemark Hospital Trust, Skien, Norway

**Keywords:** cancer, caregiver, caregiver satisfaction, early palliative care, neoplasm, next of kin, person-centered care

## Abstract

**Introduction:**

Palliative care is often introduced late in the disease trajectory. Earlier access to palliative care has been reported to benefit patient outcomes, but few studies have explored its impact on caregivers. PALLiON (PALLiative care Integrated in ONcology) was a cluster-randomized controlled trial designed to integrate systematic palliative care earlier in oncology settings. The aim of this follow-up study was to assess whether the intervention influenced caregivers’ satisfaction with care in the intervention group compared to the standard care group, and to explore whether patients’ quality of life (QoL) at inclusion influenced caregivers’ satisfaction with care over time.

**Methods:**

The trial was conducted across 12 Norwegian hospitals. The intervention included a physician education program, a patient-centered care pathway, and systematic symptom assessment. Adult patients with advanced cancer and their caregivers were recruited. Caregivers’ satisfaction with care was assessed longitudinally using the FAMCARE-20 scale, while patients’ QoL was measured with the EORTC QLQ-C15-PAL questionnaire. Linear mixed-effects regression models were used to examine the intervention’s effects, and partial least squares regression to examine associations between the intervention and responses to each FAMCARE-20 item.

**Results:**

A total of 432 caregivers of 660 patients participated between 2017 and 2021. The average caregiver age was 61.8 years (SD = 13.2), and 71% were spouses or partners. The intervention had no significant effect on caregivers’ overall satisfaction with care (*B* = -0.070, 95% CI [-0.23, 0.082], *β* = 0.02, *p* = 0.35), and the groups did not significantly differ in slope of satisfaction with care over the first 6 months (*B* = 0.040, 95% CI = [0.003, 0.08], *β* = 0.07, *p* = 0.07). Lower patient QoL at baseline was significantly associated with greater increases in caregiver satisfaction over time in both groups (*B* = 0.001, 95% CI [0.0001, 0.003], *β* = 0.06, *p* = 0.03).

**Conclusions:**

Caregivers’ satisfaction with care did not differ between the intervention and control groups. Satisfaction seemed to be more affected by patients’ baseline QoL rather than early palliative care. Thus, a systematic, person-centered approach rooted in needs may be more beneficial than a purely time-based palliative care model.

ClinicalTrials.gov: NCT03088202.

## Introduction

Cancer remains the second leading cause of death globally (1) despite advancements in treatment. At the same time, patients with advanced cancer can live with the disease for extended periods. One challenge related to increased longevity is the provision of anti-cancer treatments too late in the trajectory of their illness, with marginal efficacy. Moreover, these therapies can cause severe and persistent side effects, adding to the burden caused by both the progressive disease and emotional distress. Such side effects, in turn, can decrease patients’ health-related quality of life (QoL), increase the symptom burden and result in unplanned hospital admissions (2, 3). When patients with advanced cancer are hospitalized, their stays tend to be shorter, and their primary contact with specialist health services now occurs most frequently in outpatient settings (4). All of the aforementioned factors may result in a substantial burden for caregivers as they face greater responsibilities for longer periods.

Caregivers of patients with advanced cancer have reported unmet needs (5), limited support from the healthcare system (6, 7), and a lack of preparedness for the multifaceted role they are expected to fulfill in addition to their daily life and functions, such as caring for children or maintaining their health and employment. The caregiver role often includes monitoring side effects and disease progression, managing symptoms, providing emotional support, and assisting with personal, technical, and financial needs (8–10). Concerns regarding the current situation, uncertainty about the future, and a perceived high burden of caregiving have been shown to lead to varying degrees of distress (11, 12). High levels of distress over time may aggravate caregivers’ morbidity and impair their overall quality of life (QoL) (8). Studies suggest that the responses of patients and their caregivers to the stress and demands associated with advanced cancer are (inter)related (13). Hence, enhanced support and services for caregivers are needed to reduce their burden and improve the well-being of both caregivers and patients (14).

In recent decades, substantial evidence from randomized controlled trials (RCTs) has confirmed that palliative care delivered alongside anti-cancer therapy improves clinical and patient outcomes (15, 16). Palliative care focuses on improving QoL and reducing the suffering of seriously ill patients and their families through early identification, support, and treatment of physical, psychosocial, and spiritual problems (17). In fact, the World Health Organization (WHO) encourages the early introduction of palliative care in the management of advanced disease trajectories from the start of care, according to their needs (17). This care should be delivered through person-centered and integrated healthcare services that address the comprehensive needs of the person with the disease, rather than focusing solely on the disease itself. Such services should be offered based on both time-based and need-based criteria (3). There is evidence that some direct palliative care interventions for caregivers (e.g., psychoeducational and coping-focused interventions) positively affect various self-reported outcomes, such as QoL and mental health (14, 18). Results of research on the association between early palliative care interventions and caregiver outcomes have been mixed and have been less frequently studied using RCTs (19–21). However, qualitative studies have highlighted positive attitudes and experiences with early palliative care among both caregivers and patients (22–26).

Despite guidelines and research supporting early introduction of palliative care, referrals remain late, inconsistent, and are usually made after anti-cancer treatment has been discontinued (3). Given this background, the cluster-randomized controlled trial (C-RCT) PALLiON (Palliative Care Integrated in Oncology), was conducted in Norway from 2017 to 2021, with use and discontinuation of anti-cancer therapy during the last three months of life as its primary outcome. The C-RCT showed no statistically significant differences between the intervention and control groups for these primary endpoints (27). Although PALLiON did not include a specific caregiver program, caregiver satisfaction with patient care was a secondary endpoint (28), which is considered an important indicator of care quality (29, 30) together with QoL. In this paper, we present the caregiver outcomes from the intervention and control groups.

Based on previous literature (31, 32), we hypothesized that caregivers of patients receiving the intervention would report better satisfaction with care compared to caregivers of patients receiving standard care, and that this effect would be particularly pronounced among caregivers to patients with the lowest Qol at baseline.

We anticipated that the effects of the intervention on caregivers could manifest in two pathways: a direct effect, as palliative care inherently focuses on caregivers through its holistic, person-centered, and family-oriented approach (17); and an indirect effect via a spillover mechanism, whereby caregivers report better satisfaction with care as patients experience better symptom control and QoL (33).

The primary aim of this study was to explore differences in caregiver satisfaction with care between the intervention and control groups in PALLiON. The secondary aim was to examine whether patients’ global QoL at baseline influenced caregiver satisfaction over time when comparing the respective intervention and control hospitals.

## Materials and methods

### Study design

The current study used longitudinal patient- and caregiver-reported outcomes data from the national, parallel-group PALLiON cluster-RCT. PALLiON study details have been previously published (27).

In short, PALLiON was conducted in oncology departments with specialized palliative care programs at 12 hospitals (six intervention and six control sites) across Norway’s four health regions. The hospitals were grouped into three strata (small, medium, and large) before randomization, based on the size of their catchment areas. The sample size was 300 patients per arm, calculated based on the PALLiON primary outcome (27, 28).

### Intervention

The complex intervention in the PALLiON study was developed based on previous literature, international guidelines, and recommendations. It consisted of three components: 1) an educational program for healthcare professionals; 2) patient-centered care pathways, including early referral to palliative care; and 3) systematic symptom assessment.

**Figure 1:**
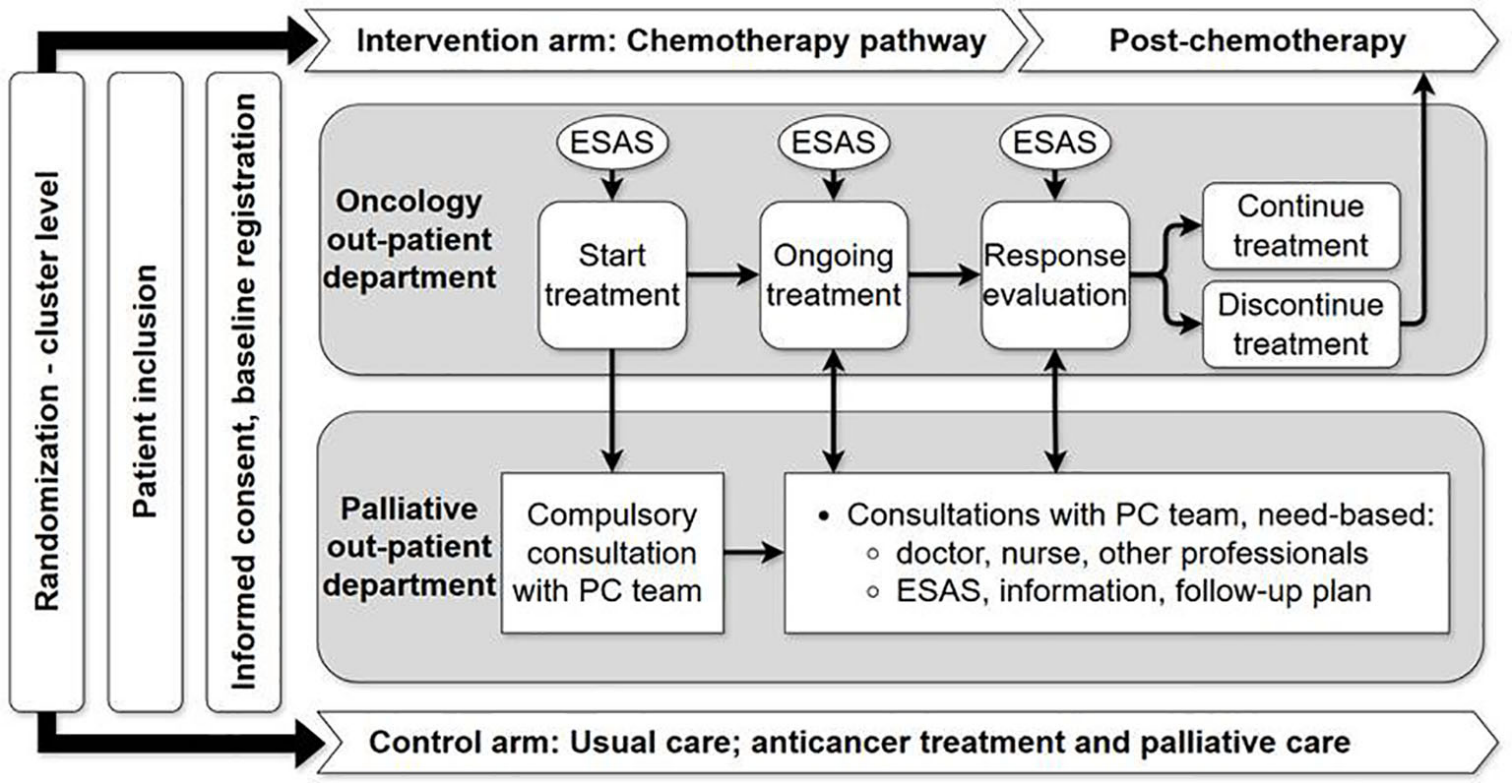
The PALLiON care pathway during anti-cancer treatment. The oncologists were responsible for cancer treatment, while specialized palliative care teams managed palliative care. Prior to consultations, the Edmonton Symptom Assessment System (ESAS) was used to register symptoms, either on paper or via the digital tool Eir.

The educational program was completed prior to the study’s initiation, while two other components of the intervention commenced upon the inclusion of the patients and their caregivers. The patient-centered care pathways (see **Figure 1**) included a mandatory referral to a specialized palliative care team consultation for all cancer patients upon initiation of their presumed last line of treatment. Further contact with the palliative care team was need-based. While there was no dedicated program for caregivers, their involvement in all consultations was emphasized as part of the intervention. Further details of the intervention are described elsewhere (28, 34).

**Figure 2:**
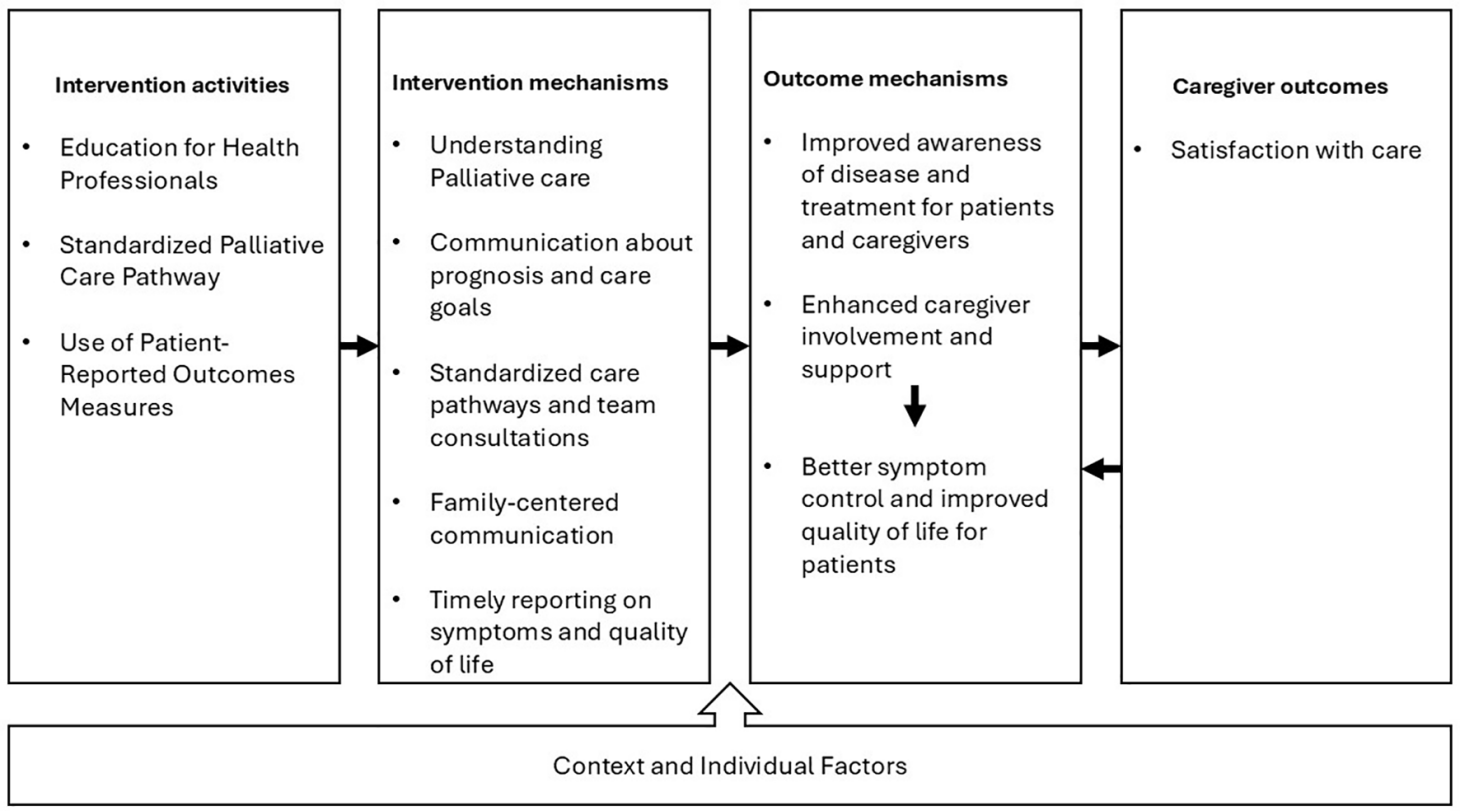
The figure illustrates a proposed logic model underlying the intervention. From left to right, intervention activities are assumed to contribute to improved knowledge and skills among healthcare professionals, as well as to the adoption of standardized care pathways and systematic assessment in clinical practice. This may facilitate increased empowerment and involvement of patients and caregivers, along with improved symptom relief through routine symptom mapping and caregiver support. An arrow from caregiver outcomes indicate a potential bidirectional influence between patients and caregivers. Contextual and individual factors are assumed to affect both the mechanisms and outcomes.

To explain the basis of our hypothesis regarding the expected effects of the intervention on caregiver outcomes, we developed a proposed logic model underlying the intervention (**Figure 2**).

### Patient and caregiver inclusion and exclusion criteria

In PALLiON, a caregiver was defined as “the person in a close, supportive role sharing the illness experience with the patient, according to the patient” (28). Patients and caregivers were recruited simultaneously. Patients were required to have an advanced-stage solid tumor, an estimated life expectancy of ≤ 12 months, and to be starting their last line of anticancer therapy. After patients had provided written consent to participate, they were asked to identify their primary caregiver. Once this consent was obtained, the identified caregiver was invited to participate by a research nurse. Inclusion criteria for both patients and caregivers were age 18 years or older, and fluency in written and spoken Norwegian. Caregivers also needed to be physically and cognitively capable of providing written informed consent. Further details regarding the study’s inclusion and exclusion criteria for the patients and caregivers can be found in the study protocol (28).

### Data collection

Self-reported data from patients and caregivers were collected at baseline and every eight weeks (using questionnaires sent by mail), up to seven times during the first year or until the patient’s death or withdrawal. At baseline, both patients and caregivers briefly reported information on sociodemographic variables. The caregivers reported: age, education level, work status, relationship to the patient, co-housing, and presence of chronic diseases. Patient’s clinical data (e.g., diagnosis, Karnofsky Performance Status (35), and treatment) were retrieved from the electronic health record journal and entered into an electronic case report form every other month.

In order to investigate the association between patients’ baseline QoL and caregivers’ satisfaction with care, one item *How will you rate your overall quality of life during the past week?* from the European Organization for Research and Treatment of Cancer Quality of Life Questionnaire Core 15 Palliative Care Questionnaire (EORTC QLQ-C15-PAL Questionnaire) (36) was used to measure patients’ global QoL. The item was scored on a 1–7 scale (very poor to excellent) and transformed to a 0–100 scale.

### Outcome measure (dependent variable)

#### Satisfaction with care

The 20-item Family Satisfaction with End-of-Life Care Scale (FAMCARE- 20 scale) (37) was used to explore whether the intervention has an impact on caregivers’ satisfaction with care. It is widely used in cancer research and has been used in RCTs in early palliative care settings (31, 32). The scale is well validated and measures caregivers’ satisfaction with the care provided to both the patients and themselves, covering areas such as information, accessibility, care coordination, and both physical and psychological care (30, 37). Scores on the FAMCARE-20 range from 1 to 5, with a score of 1 indicating the highest level of satisfaction on its five-point rating scale: 1 = Very Satisfied, 2 = Satisfied, 3 = Undecided, 4 = Dissatisfied, and 5 = Very Dissatisfied (37).

### Statistical analyses

The analyses were conducted in R version 4.4.1 (R Core Team, 2024), using the lme4 package for linear mixed-effects models (38). Categorical variables were expressed as frequencies and percentages, and continuous variables as the mean ± SD.

To examine the intervention’s effect on the caregivers’ satisfaction with care (primary aim), we used linear mixed-effect models (39) to account for the nested structure of the data, with hospitals and patients as random intercepts. The main model included time points, group assignments, and their interaction as fixed effects.

A subsequent analysis included multiple patient covariates (age, gender, cancer diagnosis code, Karnofsky Performance Status and QoL) and caregiver covariates (age, relationship to the patient, education level, work status, civil status, co-housing, and chronic illness) to explore whether controlling for these variables would alter the interpretation of the group effect. We reported parameter estimates (unstandardized regression coefficients) and their 95% confidence intervals. We used two-tailed p-value for significance, computed 95% CIs using the Wald approximation, and estimated beta coefficients (β). Standard effect sizes were used to interpret the magnitude of these effects.

To examine the potential moderating effect of patients’ global QoL at baseline (secondary aim), we estimated an extended version of the main model that also included baseline QoL of patients as a moderator of the effect of group assignment and time (a three-way interaction). Changes over time were assessed at baseline, 2, 4, and 6 months.

#### Exploratory sensitivity analyses *post hoc*

To examine whether missing data during study influenced the results, we conducted a pattern-mixture sensitivity analysis, in which participants were classified by observed missing-data pattern (complete, intermittent missingness, dropout/death). This pattern was entered into the mixed-effects model together with its interactions with time and group (40).

To assess robustness to linear change over time, we conducted a sensitivity analysis with time treated as categorical variable.

A partial least square (PLS) analysis was conducted to examine associations between the intervention and each of the 20 items of the FAMCARE-20 scale, as analyzing single-item results has been recommended in a previous study evaluating the use of the FAMCARE-20 scale (41). PLS (42) explores the multivariate associations between the 20 FAMCARE items and the intervention, allowing for exploring the joint effects of highly correlated factors on outcome. Here, we used group allocation as the dependent variable and the FAMCARE items as independent variables as recommended in a PLS-framework exploring predictors of group belonging. No covariates were included. The number of components in the PLS models was cross validated using a Monte Carlo (43) resampling with 100 repetitions. In each repetition, random samples of 50% of the caregivers were used in the calibration and validation datasets to estimate the predictive performance of each model. The minimum median root mean squared prediction error was used as a criterion to avoid over-fitting. Unstandardized coefficients *(B)* with confidence intervals were reported as all FAMCARE items have the same response categories, and confidence intervals that did not include zero were considered significant. As an example, a coefficient of 0.10 indicates that a one-point increase on the 1–5 FAMCARE item is associated with an approximately 10-percentage-point increase in the predicted probability of being in group 1 within the PLS framework. This coefficient indicates the item’s effect, but as part of the multivariate latent structure, it should not be interpreted independently of the other variables in the model.

## Results

**Figure 3:**
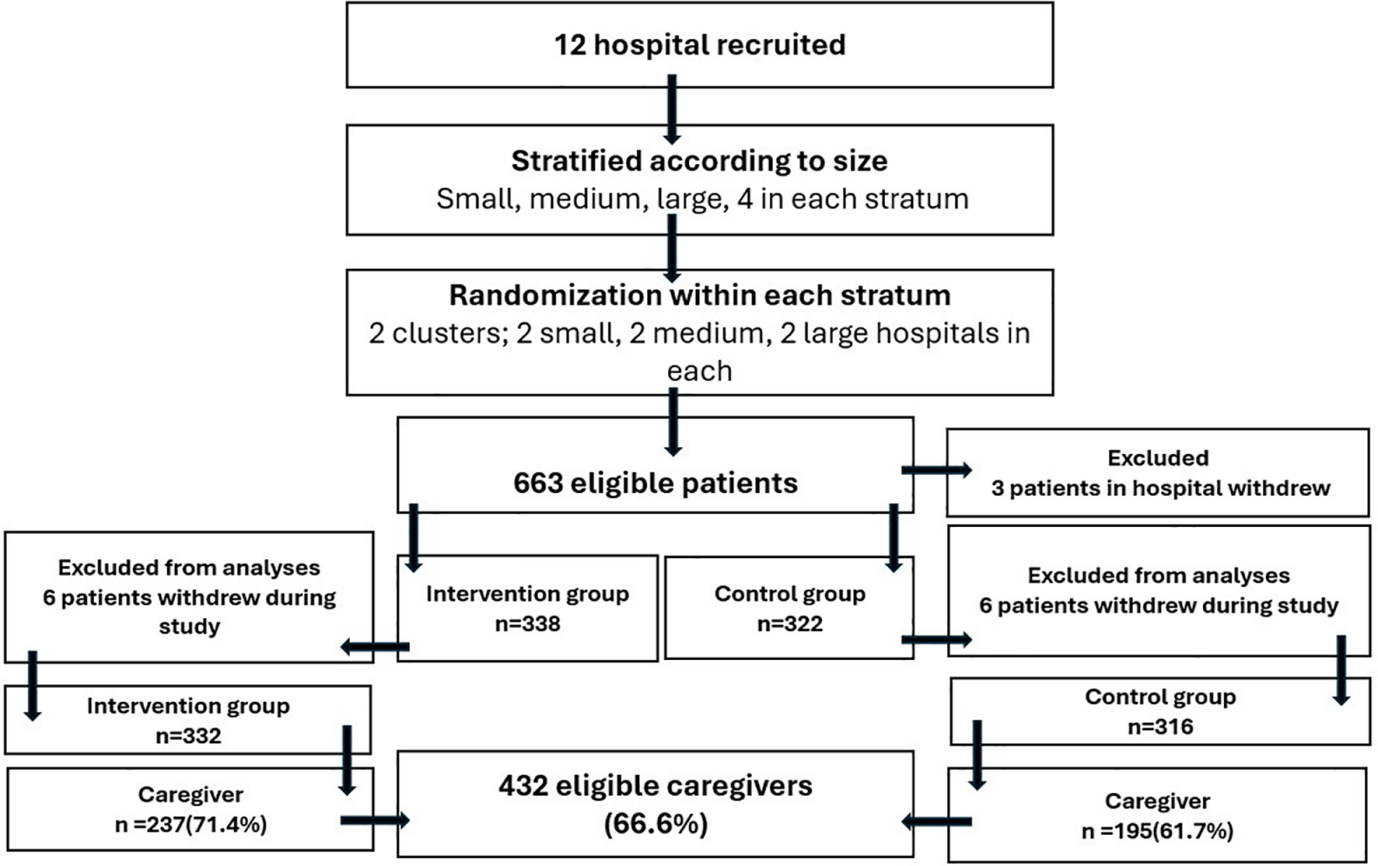
The PALLiON flow diagram, including the randomization process and the distribution of participants across the intervention and control groups.

We included 432 caregivers of the 660 patients enrolled. Overall, 66.6% of the patients in the PALLiON study identified a caregiver who consented to participate in the study (71.4% in the intervention group and 61.7% in the control group). **Figure 3** shows the caregiver flow in the PALLiON study.

### Baseline characteristics

Three hundred nine (71%) caregivers were spouses/partners. The median age of the caregivers was 61.8 years (SD 13.2) and the median age of the patients was 68.0 years (SD = 13.2). **Table 1a** presents the baseline characteristics of the caregivers, and **Table 1b** shows the patient characteristics that were relevant for our research objectives, including the patients global QoL baseline score.

**Table 1a:** Caregiver baseline characteristics of the overall sample and by groups.

Caregiver characteristics	Total (N = 432)	Usual care group (n = 195)	Intervention group (n = 237)
**Age, mean (SD)**	61.8 (13.2)	61.7 (13.5)	62.0 (12.9)
Relation to the patient %			
Spouse/partner	309 (71)	140 (72)	169 (71)
Child	85 (20)	40 (21)	45 (19)
Other relatives	22 (5.0)	11 (6)	11 (4)
Friend	4 (1.0)	1 (0)	3 (2)
Other	4 (1.0)	2 (1)	2 (1)
Missing	8 (2.0)	1 (0)	7 (3)
Shared housing with patient %			
Yes	309 (72)	139 (71)	170 (72)
No	123 (28)	56 (29)	67 (28)
Travel time to patient			
*< 30 min*	64 (15)	29 (15)	35 (15)
*≥ 30 min*	41 (9)	22 (11)	19 (8)
Missing	18 (4)	5 (3)	13 (5)
Education %			
Less than high school	62 (14)	31 (16)	31 (13)
High school	185 (43)	87 (45)	98 (41)
College	176 (41)	76 (39)	100 (42)
Missing	9 (2)	1 (0)	8 (4)
Employed %			
Yes	183 (42)	83 (43)	93 (39)
No*	249 (58)	112 (57)	144 (61)
Sick leave %			
Yes	30 (16)	9 (11)	21 (23)
No	153 (84)	74 (89)	72 (77)
Chronic illnesses %			
Yes	275 (64)	128 (65)	147 (62)
No	149 (34)	66 (34)	83 (35)
Missing	8 (2)	1 (1)	7 (3)

SD, Standard deviation *includes retirees.

**Table 1b:** Patients’ baseline characteristics of the overall sample and by groups.

Patient characteristics	Total (N = 432)	Usual care group (n = 195)	Intervention group (n = 237)
**Sex n (%)**			
Male	273 (63)	151 (77)	122 (52)
Female	158 (36)	43 (23)	115 (48)
Missing	1 (1)	1 (1)	
**Age, median (SD)**	68.0 (13.2)	67.4 (13.0)	68.7 (13.5)
**Comorbidities %**			
Yes (%)	214 (49.5)	119 (61)	95 (40)
No	218 (50.0)	76 (39)	141 (60)
Missing	2 (0.5)	1 (0)	1 (0)
Primary cancer diagnosis %			
Prostate	32 (7)	13 (7)	19 (8)
Gastrointestinal*	301 (70)	129 (66)	172 (72)
Breast	16 (4)	10 (5)	6 (3)
Melanoma	11 (3)	7 (4)	4 (2)
Other	43 (9)	26 (13)	17 (7)
Missing	29 (7)	10 (5)	19 (8)
Karnofsky performance status scale (%)			
100-80	298 (69)	134 (69)	164 (69)
70-50	124 (29)	58 (30)	66 (28)
40-0	10 (2)	3 (1)	7 (3)
Global quality of life at first timepoint mean (SD)* *	55.7 (24.9)	55.5 (25.5)	55.9 (24.5)

*Gastrointestinal cancers include stomach, esophageal, cholangiocarcinoma, pancreatic and colorectal cancers * *Measured with the EORTC QLQ-C15-PAL. Higher scores (0-100) indicate a better quality of life. Abbreviations: SD, Standard deviation.

### Differences in global satisfaction with care between the groups

**Figure 4:**
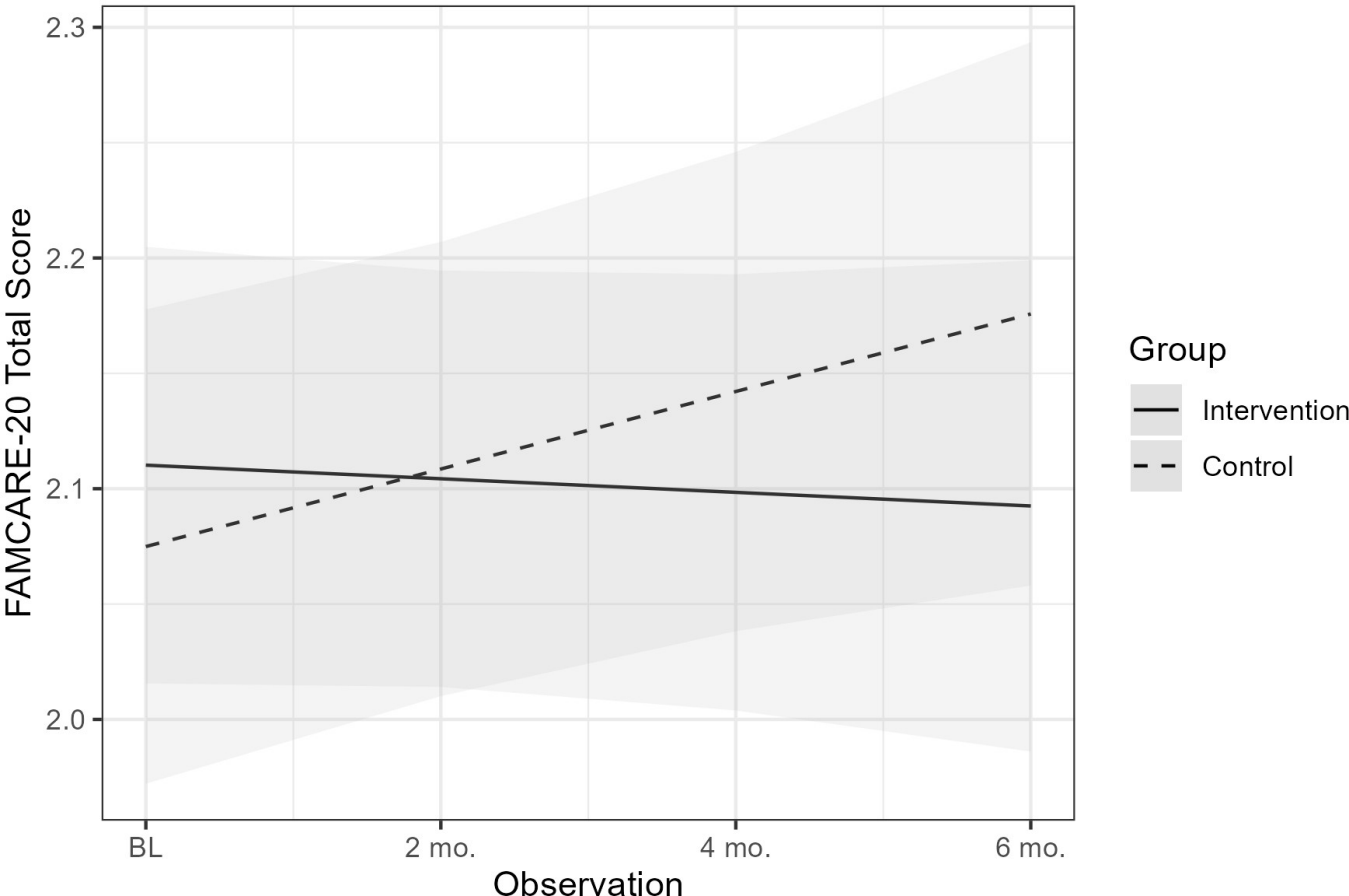
Estimated slopes of satisfaction with care (FAMCARE) over time derived from the main model. Of note, lower values indicate better satisfaction with care. The slopes did not show a statistically significant difference between the groups, *p* = 0.07.

The caregivers’ mean level of satisfaction with care was 2.08 (SD = 0.64). At baseline, 50.4% of the caregivers scored between 1–2 on the total satisfaction with care (very satisfied to satisfied), while only 1.2% scored between 4-5 (dissatisfied to very dissatisfied). No main effect of group (*B* = -0.070, 95%CI = [-0.23, 0.082], *β* = 0.02, *p* = 0.35), or interaction of group and time point was observed (*B* = 0.040, 95%CI = [0.003, 0.08], *β* = 0.07, *p* = 0.07) (**Table 2**). **Figure 4** shows the slope of the mean satisfaction with care at different points in time.

**Table 2:** Group and time point effects on caregivers’ global satisfaction with care.

Parameter	Unstandardized coefficient (*B*)	95% CI	Standardized coefficient (β)	*P*
Fixed				
Intercept	2.12	[2.01, 2.22]	0.03	<.001
Time	-0.0059	[- 0.03, 0.02]	- 0.01	0.68
Group (Control)	-0.070	[- 0.23, 0.08]	0.02	0.35
Time*Group	0.040	[0.003, 0.08]	0.07	0.07
**Random**	Variance component	SD	ICC	
Caregiver	0.290	0.54	0.68	
Site	0.004	0.06	0.009	
Residual	0.130	0.36		

Linear Mixed-effects Model, main model. Abbreviation: CI, Confidence interval, ICC, Intraclass correlation coefficient, SD, Standard deviation. The reference group was Intervention group. Time was used as a continuous variable (baseline, 2 months, 4 months and 6 months). Satisfaction with care measured with FAMCARE-20 scale. Of note, lower satisfaction values reflect greater satisfaction with care.

The subsequent analysis, which adjusted for key patient and caregiver characteristics, including patient quality of life, presence of chronic illnesses among caregivers and caregiver relation to the patient, found no overall group differences. However, presence of chronic illnesses were generally associated with worse caregiver satisfaction with care (*B* = 0.050,95%CI= [0.01, 0.10], *β =* 0.12, *p* = 0.015 whereas higher patient global QoL was associated with better satisfaction with care -0.0028,95%CI = [0.00, 0.00], *β* = -0.11, *p* =<0.001. No other covariates were significantly associated with caregiver satisfaction (**Table 3**).

**Table 3:** Results of subsequent analysis of caregiver satisfaction.

Parameter	Unstandardized coefficient (*B*)	95% CI	*β*	*P*
Fixed				
(intercept)	2.30	[1.64, 2.96]	0.36	<.001
Time	- 0.01	[- 0.04, 0.03]	-0.01	0.755
Group (Control)	- 0.10	[- 0.28, 0.09]	-0.08	0.297
Time * Group	0.04	[-0.01, 0.09]	0.07	0.144
Patient variables				
Age	0.0008	[-0.01, 0.01]	0.01	0.843
Sex	- 0.09	[-0.23, 0.04]	-0.07	0.167
Primary Diagnosis: Gastrointestinal	0.17	[-0.13, 0.47]	0.12	0.273
Primary Diagnosis: Melanoma	- 0.10	[-0.56, 0.36]	-0.03	0.666
Primary Diagnosis: Prostate	0.040	[-0.34, 0.42]	0.02	0.827
Primary Diagnosis: Other cancer	0.16	[-0.19, 0.51]	0.08	0.374
Karnofsky Performance Status	0.0011	[0.00, 0.00]	0.02	0.457
Global Quality of Life	- 0.0028	[0.00, 0.00]	-0.11	**<.001**
Caregiver variables				
Age	-0.0034	[-0.01, 0.00]	-0.07	0.386
Relationship: Spouse/significant other	-0.030	[-0.25, 0,20]	-0.02	0.811
Relationship: Other relative	0.10	[-0.20, 0.41]	0.04	0.496
Relationship: Friend	0.17	[-0.46, 0.79]	0.03	0.596
Relationship: Other	-0.34	[-0.91, 0.23]	-0.06	0.236
Education: High School	-0.13	[-0.31, 0.04]	-0.11	0.141
Education: College/University	-0.090	[-0.28, 0.09]	-0.07	0.338
Employed:	0.020	[-0.09, 0.13]	0.02	0.727
Number of illnesses	0.050	[0.01, 0.10]	0.12	**0.015**
**Random**	Variance component	SD	ICC	
Caregiver	0.251	0.50	0.651	
Site	0.009	0.09	0.023	
Residual	0.125	0.35		

Linear Mixed-effects Model, adjusted. Global Quality of life and Karnofsky Performance Status were treated as continuous, time-varying covariates; Time, age, and chronic illnesses were included as continuous covariates (baseline, 2 months, 4 months and 6 months). The reference group was Intervention group; the reference group for the primary cancer diagnosis was Breast Cancer; the reference group for the Relationship was Child; and the reference group for Education was less than high school. Satisfaction with care measured with FAMCARE-20 scale. Of note, lower satisfaction values reflect greater satisfaction with care. Global Quality of Life measured with EORTC QLQ-C15-PAL. Higher scores (0-100) indicate a better quality of life. CI, Confidence interval; ICC, Intraclass correlation coefficient; SD, Standard deviation.

### Quality of life and global satisfaction with care

Findings from the extended moderating effect model, showed that a higher baseline patients’ global QoL score at baseline was consistently significantly associated with greater caregiver satisfaction with care (B = -0.006, 95%CI = [-0.01, -0.002], β = -0.12, p = 0.002). The three-way interaction between patients’ baseline QoL, time, and group was not significant (B = 0.001, 95%CI = [-0.001, 0.003], β = 0.05, p = 0.23), indicating no significant differences in the groups’ slopes over time as a function of the patients’ baseline QoL. The two-way interaction between QoL and group was also non-significant (B = -0.002, 95%CI = [-0.007, 0.004], β = 0.03, p = 0.60), see **Table 4**.

**Table 4:** Patients’ baseline QoL as predictor of caregivers’ satisfaction with care, by group and time.

Parameter	Unstandardized coefficient *B*	95% CI	*β*	*P*
Fixed				
Intercept	2.50	[2.24, 2.76]	0.06	<.001
Time	-0.08	[-0.17, 0.0003]	-0.006	0.051
Group (Control)	-0.04	[-0.41, 0.34]	-0.07	0.847
QoL	-0.006	[-0.01, -0.002]	-0.12	**0.002**
Time * Group	-0.03	[-0.14, 0.09]	0.06	0.648
Time * QoL	0.001	[0.0001, 0.003]	0.06	**0.033**
Group * QoL	-0.002	[-0.007, 0.004]	0.03	0.602
Time *Group*QoL	0.001	[-0.001, 0.003]	0.05	0.234
	Variance			
**Random**	component	SD	ICC	
Caregiver	0.27	0.52	0.66	
Site	0.009	0.10	0.02	
Residual	0.13	0.36		

Extended model exploring potential moderating effect of patients’ global QoL at baseline. QoL was measured with the global item from EORTC QLQ-C15-PAL. Higher scores (0-100) indicate a better quality of life. Satisfaction with care measured with FAMCARE-20 scale. Of note, lower satisfaction values reflect greater satisfaction with care. The reference group was intervention group CI, Confidence interval, ICC, Intraclass correlation coefficient, SD, Standard deviation.

**Figure 5:**
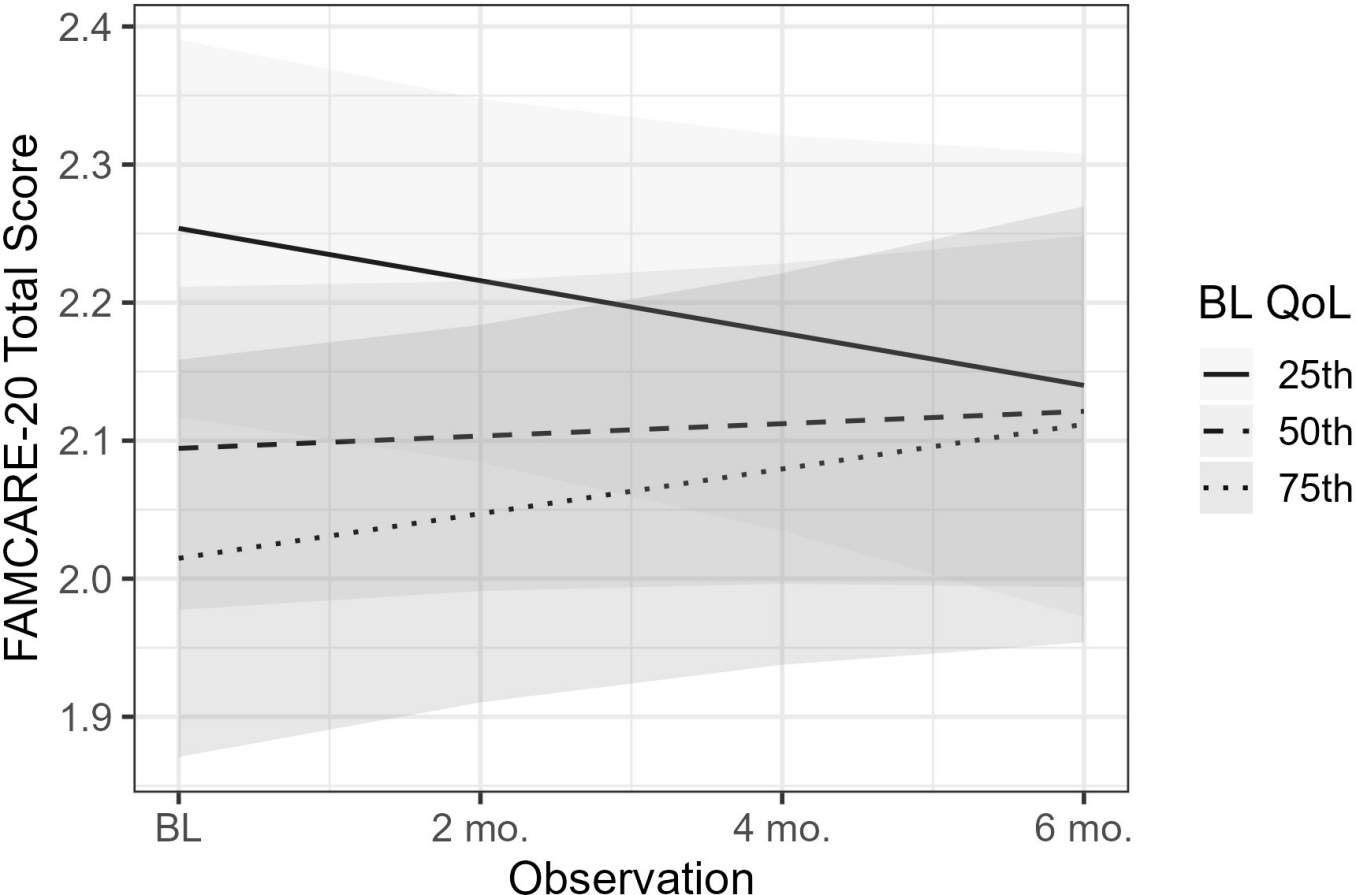
The figure illustrates differences in caregivers’ satisfaction with care (FAMCARE) over time, based on the patients’ baseline QoL. QoL is grouped into three percentiles categories: low (25^th^),medium (50^th^), and high (75^th^). Of note, lower satisfaction scores reflect greater satisfaction with care.

However, patients’ baseline QoL did significantly interact with time in predicting caregivers’ satisfaction with care (two-way interaction B = .001, 95% CI [.0001,.003], β = 0.06, p = 0.03), indicating that trajectories of satisfaction differ according to patients baseline QoL. Specifically, the analysis revealed that the caregivers of patients with a lower baseline QoL reported significantly greater increases in satisfaction with care over time in both groups, compared with caregivers of patients with a higher baseline QoL who showed a slight decrease over time. To facilitate interpretation of this unexpected finding, patients were descriptively categorized into three percentile-based groups (low, medium, or high) according to baseline QoL scores, for illustrative purposes only (**Figure 5**).

The number of observations decreased in both groups at each time point (**Supplementary Figure 1**), which corresponds with the reported median survival time from inclusion to death, which was 8 (IQR 3-14) and 7 (IQR 3-12) months (intervention/control) in the main PALLiON paper (27). The pattern-mixture sensitivity analysis indicated that the results were not affected by missing data patterns. The time-by-group effect estimate was somewhat larger in this model (B = 0.050, 95%CI=[0.00, 0.10], β = 0.08, p = 0.04) compared to the original model without missing data accounted for (B = 0.040, 95%CI=[0.003, 0.08], β = 0.07, p = 0.07). Importantly, there was no strong indication that this effect differed across missing-data patterns, as the corresponding three-way interaction terms were small and not suggestive of a substantial moderation effect (B = -0.04, 95%CI= [-0.18, 0.11], β = -0.06, p = 0.62 for intermittent missingness; B = -0.083, 95%CI=[-0.20, 0.03], β = -0.14, p = 0.16) for dropout).(**Supplementary Table 1**).

The exploratory sensitivity analysis treating time as a categorical variable did not differ from the main analysis, but showed a small mostly significantly difference (B =0.130, 95%CI [-0.007, 0.004], β =0.2, p=0.06) in favor of the intervention group at time point 3 (**Supplementary Table 2**).

### Satisfaction with care and group differences in single-item scores

**Figure 6:**
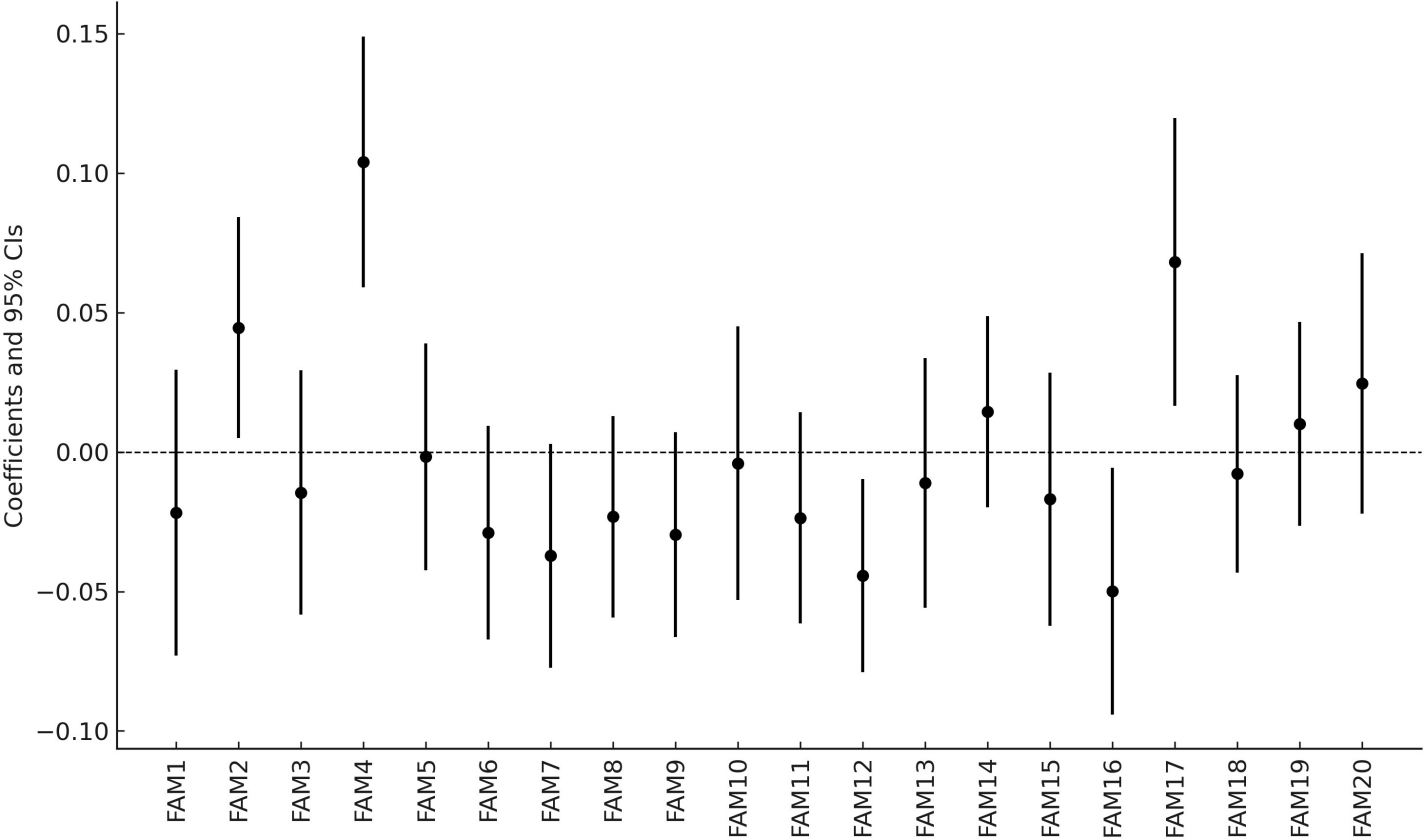
PLS regression with coefficients and 95% confidence intervals. Negative FAMCARE item values are in favor of the intervention group, and positive FAMCARE item values are in favor of the control group. Confidence intervals not including zero are considered significant.

The PLS regression model explained 4.98% of the variance of group assignment when analyzing patterns for individual FAMCARE-20 items favoring either the intervention or control group (aggregated across all time points). The results from this exploratory sensitivity analysis indicated items that exhibited small but significant differences between the groups. Two single-item scores showed greater satisfaction with care in the intervention group: *Availability of nurses to the family* (item 12) and *Information about how to manage the patient’s pain* (item 16). The control group rated satisfaction with care better on three single items compared to the intervention group: *Information provided about the patient’s prognosis* (item 2); *Information given about side effects* (item 4); and *Information given about the patient’s tests* (item 17). The remaining items had wider confidence intervals, including 0, indicating no significant differences (see **Figure 6**).

## Discussion

Overall, caregivers in both groups reported to be satisfied with the care at baseline and throughout the study period. The findings showed no statistically significant group differences in the interactions on caregivers’ global satisfaction with care. The subsequent analysis did not provide evidence of an intervention effect at the group level when adjusted for patient and caregiver covariates. However, the covariate analyses provide important insight into factors that may influence caregiver satisfaction with care independently of group assignment and time. Caregivers with chronic illnesses were associated with worse satisfaction with care, whereas caregivers of patients with higher QoL were associated with greater satisfaction with care. Although effect sizes were small, these findings may be clinically relevant at the individual level. Caregivers who experience reduced health themselves have been found to be less resilient to the burden associated with the caregiving role (44). Our findings suggest that these caregivers may need closer support to achieve satisfaction with care.

We found also evidence for an association between higher patient QoL and greater caregiver satisfaction in an extended version of the main model, in which patient’s baseline QoL alone demonstrated moderating effect on the caregiver satisfaction’s trajectories. This association is consistent with findings from a cross-sectional study by Hannon et al. (45), which also used FAMCARE scale to examine satisfaction with care among cancer caregivers.

The extended model analysis yielded two particularly interesting findings. 1. We found no differences in caregiver satisfaction trajectories between the intervention and control groups based on patients baseline QoL, contrary our hypothesis. Instead, low patient baseline QoL appeared to be a factor that affected caregivers’ satisfaction in the same way, regardless of group allocation. 2. Caregivers of patients with a low baseline QoL in both groups, generally became more satisfied with care over time, whereas caregivers of patients with a high baseline QoL experienced a weak decrease in satisfaction over time. Overall, this trend appeared to result in largely comparable levels of satisfaction among caregivers at the 6-month follow-up, regardless of patients’ baseline scores. This effect was small and should not therefore be over-interpreted, but it may be relevant given the expected benefits of early palliative care should accrue especially to those patients with low QoL to begin with. The significant differences in this outcome may be explained by the fact that the patients with the lowest baseline QoL in the control group received a certain degree of relief and support, which proved sufficient to enhance the caregivers’ satisfaction with the care provided. The observed increase in satisfaction with care may reflect a sense of gratitude (46), rather than a genuine improvement in care quality, as satisfaction is a multidimensional construct influenced by factors such as expectations, health status, and socioeconomic and demographic conditions (29). Alternatively, the generally high baseline scores of satisfaction with care in our study may genuinely reflect the quality of the national health care in Norway, as experienced by patients and caregivers.

A few significant differences in single-item scores were observed between groups, but the overall explained variance was low (47). Item-level effect sizes were small, mostly close to zero, and of limited practical significance. They were also inconsistent in direction, with some favoring the intervention group and others the control group. Furthermore, it is important to emphasize that this analysis was exploratory, aimed solely at identifying patterns, and should therefore be interpreted with caution.

Our main findings both contrast with and align with results from other RCTs in early palliative care, which, like our study, did not include a specific caregiver program. A Canadian cluster-RCT by McDonald et al. (32) found an increased caregiver satisfaction with care in the intervention group compared to that in the control group. Although the caregivers in that study had similarly high total FAMCARE baseline scores as ours, the researchers found a significant positive intervention effect at three and four months. However, consistent with patterns identified in our study, they observed that certain items from the FAMCARE- 20 scale were affected more than others. One of these items, *Availability of nurses to the family*, was consistent with the findings from our exploratory PLS analysis, which was one of two items on which caregivers in the intervention group scored better.

In a Belgian RCT, Vanbutsele et al. (48) found, like us, no differences in caregiver satisfaction with care, as measured with the FAMCARE-20, between intervention and control groups— neither in total score nor subscales. In a C-RCT conducted in Australia and the UK (49), caregivers in the intervention group reported greater overall satisfaction with care, which contrasts with our findings. Yet, some findings aligned with ours in that caregivers in the intervention group were more satisfied with *the attention to the patient’s symptoms* and *the emotional support to family members*. Since a revised version of the FAMCARE- 20 scale questionnaire was used in that study (the 17 item FAMCARE-2 scale), combined with a different intervention design and a distinct diagnostic group (malignant pleural mesothelioma), direct comparisons remain challenging.

Exceeding an already high level of caregivers’ satisfaction with care is a prerequisite for detecting additional improvements, a phenomenon that has been previously discussed in the context of ceiling effects in palliative care research (31, 32). The sensitivity of the FAMCARE-20 scale in our study outcomes warrants scrutiny. Although FAMCARE-20 is recognized as a valid and reliable tool for measuring end-of-life-quality of care (37), ceiling effects and healthy user bias present challenges in evaluating interventions and identifying dissatisfaction with care, which should be considered when interpreting results (29). Despite these challenges, several individual items appeared clinically valuable, and the single-item scores may offer a better basis for comparisons with other studies and highlighting caregiver dissatisfaction, which is important for developing better healthcare services (41).

Other factors may also have contributed to the lack of statistically significant effect of the three-part intervention on caregivers. First, no intervention effect was found for the primary outcome in PALLiON (use of anti-cancer therapy in the last 3 months), nor for patient-reported outcomes (27). Therefore, the findings of our study were not surprising considering the cascading effect hypothesis, which suggests that a patient’s well-being or dissatisfaction may influence caregiver’s outcomes similarly, consistent with studies suggesting that couples respond as an emotional system (33, 50).

Second, PALLiON was powered only for the main primary outcome on which our analyses are not based. This may explain why we did not observe statistically significant differences on other measures.

Third, a key challenge in implementing complex interventions in real-world settings is ensuring full adherence to the intended protocol. A small process evaluation conducted at one intervention hospital in PALLiON assessed clinician adherence to the protocol during oncological and palliative care consultations (51). The evaluation addressed if four core indicators (ESAS, Karnofsky Performance Status, body weight, and a written summary to the patient’s general practitioner) were performed in 76 patients, covering 435 consultations. These indicators were mandatory to document in the patient record at all consultations. The findings indicated variable clinician adherence to protocol components, and that the registrations varied across consultations and fluctuated over time. Mean adherence reached 94.8% in the palliative consultations, 65.8% in first oncological consultation, and 69.2% in consultations during anticancer therapy. Assessments of caregiver issues were not mandatory, however recommended to address and document in palliative care consultations. The process evaluation included patients only.

Adherence is a universal problem in clinical studies, where the exact operating procedures from pharmaceutical trials are often missing. These findings highlight the challenge of changing practice even in a well-supported clinical trial and are consistent with other studies (52–54).

As a final consideration, questions arise as to whether the intervention was strong enough to produce a change in clinical practice that impacts caregivers, particularly in the absence of specific interventions and follow-up directed at caregivers. We argued that the planned intervention may have both indirect and direct effects on caregivers: indirectly through a spillover effect arising from the patient’s response to the intervention, and directly because palliative care inherently focuses on caregivers through its holistic, person-centered, and family-oriented approach (17). This presupposes that the intervention has been implemented as intended and that earlier access to palliative care is accepted and desired by patients and their caregivers. On the one hand, the patients’ prognosis was less than one year when they started their last line of treatment, suggesting that patients and their caregivers might benefit from earlier access to palliative care. On the other hand, there is a strong societal stigma associated with palliative care as end-of-life care (55, 56), which may have influenced patients’ and caregivers’ readiness for a palliative approach while initiating anticancer therapy. Even though early referral to palliative care is generally regarded as more beneficial than late referral, the ideal timing for initiating such care remains undefined and subject to ongoing discussion (57–59). Nevertheless, a clear consensus in referral guidelines emphasizes the provision of early palliative care, ideally based on a combination of both time-based and need-based criteria (3, 60). Our findings in this study may support such a model.

A strength of this study is the inclusion of a substantial number of participants, representing 12 different hospitals across Norway. In addition, the longitudinal C-RCT design, with several measurement points, made it possible to monitor caregivers’ satisfaction with care over six months. A limitation of this study is the unequal distribution of diagnostic groups, with a significant overrepresentation of caregivers to patients with gastrointestinal diagnoses. The process evaluation indicated variable clinician adherence to the protocol components, i.e. a suspicion of low fidelity. In retrospect, this is a potential limitation in the design of the PALLiON study, which was planned back in 2015. Had the study been developed according to current standards, a stronger emphasis on ensuring proper implementation of the intervention might have prevented a negative study conclusion due to low fidelity. Furthermore, although caregiver outcomes were defined only as secondary outcomes, it became apparent afterwards that the lack of assessing caregiver burden or needs also represented a limitation. Such measures could have provided more comprehensive information about the caregivers’ situation and thereby facilitated the interpretation of our findings.

Future studies should: 1) explore the impact of integrating early palliative care in oncology through interventions that include a structured assessment of caregiver burden and needs; 2) provide tailored support based on those assessments; and 3) evaluate caregiver satisfaction with care throughout the cancer trajectory. This approach could support a more person-centered model of care for caregivers, and provide insight into whether satisfaction with care correlates with caregivers’ own needs and caregiving-related burden. Qualitative interviews, in addition to questionnaires to caregivers, could have provided complementary data to better highlight caregivers experiences and, guide future studies.

In conclusion, satisfaction with care among the PALLiON study caregivers did not differ significantly between the intervention and control groups. In this sample, caregivers’ satisfaction with care over time seemed to be more directly influenced by patients’ baseline QoL than by the early palliative care intervention. This finding suggests that a systematic, person-centered approach may be more beneficial than an approach purely based on a time model for allocating specialized palliative care resources.

## Data Availability

The dataset presented in this article is not readily available because the study's ethical approval does not permit public sharing of the data. Requests to access the dataset should be directed to the corresponding author.
