# Supplementary material for: Caregiver satisfaction with early integrated palliative care in oncology: secondary outcomes from the PALLiON cluster-RCT
Source: Front Oncol. 2026 Jun 18;16:1787814. doi: 10.3389/fonc.2026.1787814 (PMC13322938; doi:10.3389/fonc.2026.1787814)
Supplement: Supplementary file 1 [file Table1.docx]

**Supplementary Table S1. Pattern-mixture model.**

| Parameter | Unstandardized coefficient (*B*) | 95% CI | Standardized coefficient (β) | *p* |
| --- | --- | --- | --- | --- |
| **Fixed** |  |  |  |  |
| (Intercept) | 2.05 | [ 1.90, 2.19 ] | -0.11 | <.001 |
| Time | -0.01 | [- 0.04, 0.02 ] | -0.02 | 0.39 |
| Group (Control) | -0.02 | [- 0.24, 0.20 ] | 0.14 | 0.85 |
| Pattern (Int.) | -0.14 | [- 0.51,  0.22 ] | -0.01 | 0.44 |
| Pattern (Drop) | 0.12 | [ -0.08,  0.32 ] | 0.32 | 0.24 |
| Time *Group (control) | 0.05 | [ -0.00,  0.10 ] | 0.08 | 0.04 |
| Time *Pattern (Int.) | 0.06 | [ -0.03, 0.15 ] | 0.1 | 0.18 |
| Time *Pattern (Drop) | 0.04 | [ -0.03, 0.12 ] | 0.07 | 0.29 |
| Group (Control)*Pattern (Int.) | 0.31 | [ -0.27, 0.89 ] | 0.36 | 0.29 |
| Group (Control)*Pattern (Drop.) | -0.07 | [ -0.37, 0.23 ] | -0.39 | 0.65 |
| Time*Group (control)*Pattern (Int.) | -0.04 | [ -0.18, 0.11 ] | -0.06 | 0.62 |
| Time*Group (control)*Pattern (Drop) | -0.08 | [ -0.20, 0.03 ] | -0.14 | 0.16 |
| **Random** | Variance  component | SD |  |  |
| Caregiver | 0.289 | 0.538 |  |  |
| Site | 0.004 | 0.061 |  |  |
| Residual | 0.124 | 0.352 |  |  |

*Note*. Abbreviation: CI, Confidence interval, ICC, Intraclass correlation coefficient, SD, Standard deviation, Int., Intermittent missingness, Drop., Dropout/death. The reference group was Intervention group for the Group coefficient and Complete data for the missing data patterns.
