# Supplementary material for: Caregiver satisfaction with early integrated palliative care in oncology: secondary outcomes from the PALLiON cluster-RCT
Source: Front Oncol. 2026 Jun 18;16:1787814. doi: 10.3389/fonc.2026.1787814 (PMC13322938; doi:10.3389/fonc.2026.1787814)
Supplement: Supplementary file 2 [file Table2.docx]

**Supplementary Table S2. Group and time point effects on caregivers’ global satisfaction with care with time as categorical variable.**

| Parameter | Unstandardized coefficient (*B*) | 95% CI | t(1175) | Standardized coefficient (β) | *p* |
| --- | --- | --- | --- | --- | --- |
| **Fixed** |  |  |  |  |  |
| Intercept | 2.09 | [   2.00, 2.19 ] | 42.33 | 0.01 | <.001 |
| Time 1 | 0.05 | [- 0.03, 0.12 ] | 1.24 | 0.08 | 0.214 |
| Time 2 | 0.007 | [- 0.08,  0.09 ] | 0.16 | 0.01 | 0.876 |
| Time 3 | -0.01 | [- 0.10,  0.08 ] | -0.3 | -0.02 | 0.764 |
| Group (Control) | -0.01 | [ -0.15,  0.13 ] | -0.15 | -0.02 | 0.879 |
| Group*Time 1 | -0.030 | [ -0.14,  0.09 ] | -0.46 | -0.04 | 0.648 |
| Group*Time 2 | 0.030 | [ -0.10,  0.15 ] | 0.43 | 0.04 | 0.666 |
| Group*Time 3 | 0.130 | [   0.00, 0.27 ] | 1.91 | 0.2 | 0.057 |
| **Random** | Variance  component | SD | ICC |  |  |
| Caregiver | 0.290 | 0.54 | 0.68 |  |  |
| Site | 0.004 | 0.06 | 0.009 |  |  |
| Residual | 0.131 | 0.36 |  |  |  |

Abbreviation: CI, Confidence interval, ICC, Intraclass correlation coefficient, SD, Standard deviation. Time; Baseline (reference). Group; Intervention (reference). Satisfaction with care measured with FAMCARE-20 scale. Of note, lower satisfaction values reflect greater satisfaction with care.
