# Supplementary figures and images for: Caregiver satisfaction with early integrated palliative care in oncology: secondary outcomes from the PALLiON cluster-RCT
Source: Front Oncol. 2026 Jun 18;16:1787814. doi: 10.3389/fonc.2026.1787814 (PMC13322938; doi:10.3389/fonc.2026.1787814)

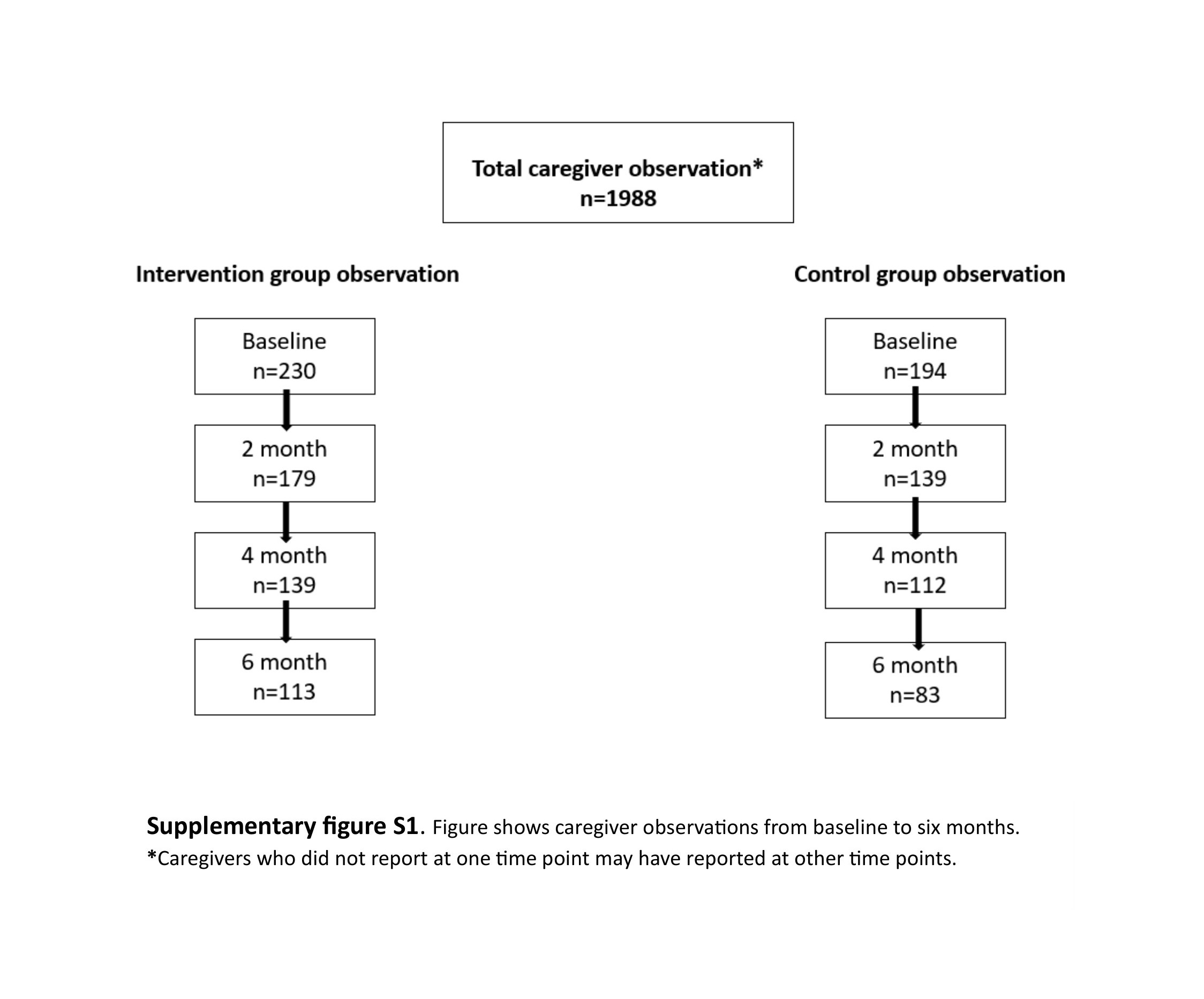

Supplement: Supplementary file 3 [file Image1.jpeg]
